# Supplementary material for: Association Mapping for Evaluation of Population Structure, Genetic Diversity, and Physiochemical Traits in Drought-Stressed Maize Germplasm Using SSR Markers
Source: Plants (Basel). 2023 Dec 7;12(24):4092. doi: 10.3390/plants12244092 (PMC10747078; doi:10.3390/plants12244092)
Supplement: Supplementary file 1 [file plants-12-04092-s001.zip › Table S1.pdf]

Table S1. Seven physiochemical traits of 41 drought-stressed maize inbred lines.

Inbred lines	Caffeic acid (µg/mL)	Chlorogenic acid (µg/mL)	Gallic acid (µg/mL)	Ferulic acid (µg/mL)	TPC (mg GAE/g sample)	DPPH (%)	LRWC (%)
11BS8016-7	0.00000	2.21444	3.36412	1.62292	87.49635	17.01913	64.85223
12BS5076-8	1.37209	0.00000	2.18342	0.00000	125.89051	4.83384	64.39238
12S8052	2.12573	1.23227	2.92662	1.47435	179.19951	12.68882	64.08335
14S8025	1.16181	1.41328	4.66789	1.46052	136.35280	18.73112	69.95716
15RS8039	1.93677	1.38761	2.47247	1.12888	110.00243	15.50856	72.52193
15RS8056	1.32595	2.36475	3.19200	1.37683	134.96594	22.75932	69.59681
15RS8002	0.96227	2.17231	2.35043	0.00000	88.00730	12.61803	68.26427
15S8021-3	2.79434	4.41830	4.25498	1.50752	142.97080	9.76253	72.39984
16CLP23	2.91563	1.91358	4.94876	1.47657	148.71290	0.00000	76.81975
16CLP40	2.45734	3.01391	4.73313	1.42004	131.77859	0.00000	74.61175
17CS5047	1.46554	0.87104	4.01785	1.27740	83.74939	0.00000	77.76660
16S8068-9	1.59761	3.17125	4.82098	1.23318	99.36983	1.65347	65.67475
17CS8006	0.91810	2.99948	5.41431	0.97855	81.26764	0.29903	67.04267
17CS8067	0.64195	1.17894	2.44530	0.63772	60.75669	0.00000	68.00526
17YS6032	0.83017	3.28306	3.24804	1.02117	103.74939	0.00000	71.21341
17YS8003	1.14969	1.23415	3.53246	1.01149	111.14599	0.00000	68.55942
GP3	1.30489	4.13404	2.06470	1.20441	151.72993	0.00000	73.13835
GP5	1.61642	3.79061	3.15867	1.27642	132.04623	0.00000	71.70087
HCW1	0.69275	2.62817	3.95160	1.11178	108.51825	0.00000	75.75835
HCW2	1.15836	1.39747	5.00942	1.83362	118.63990	0.00000	76.46382
HCW3	1.09932	4.50064	3.66804	1.39421	96.69343	0.00000	65.41318
HCW4	0.76620	1.94388	4.84241	0.74422	100.61071	0.00000	73.84913
HCW5	1.01920	3.03165	3.56124	1.14270	116.66910	1.53034	72.58081
HF12	0.68420	0.85761	3.55011	0.75376	80.63504	0.00000	75.86548
HF22	0.91842	1.56856	3.10974	1.10614	99.88078	0.00000	70.99745

HW1	0.73946	1.53641	5.10001	1.38820	91.77859	0.00000	70.67959
HW10	1.09144	0.00000	3.93971	1.43453	84.86861	0.00000	68.66205
HW11	1.53167	0.98838	3.69988	0.70379	55.11192	0.00000	68.69350
HW12	1.01474	2.21908	4.24190	1.06603	74.04136	1.20172	66.43930
HW15	0.75634	0.00000	3.77956	0.78281	80.48905	1.97425	53.08914
HW16	0.96223	1.67211	2.88110	0.89045	86.98540	0.68670	69.35619
HW17	1.61899	1.42745	3.08932	1.16220	92.21655	1.08727	70.41692
HW18	1.05455	2.79576	2.45533	0.00000	82.63017	0.00000	72.10552
HW19	1.17983	5.12761	2.78129	1.02395	130.09976	0.00000	68.38966
HW3	1.64625	1.24375	3.73139	1.06577	95.28224	1.54506	66.80877
HW4	1.92070	2.28064	3.32507	1.24229	108.95620	5.41116	64.96052
HW7	1.87270	0.00000	4.18404	1.79994	100.48905	2.59906	65.55221
HW8	0.99560	1.55383	3.33928	0.80114	79.56448	10.60929	67.01877
HW9	1.17311	1.76672	2.38340	0.00000	63.23844	0.96577	64.44524
KL103	1.38483	0.00000	4.65354	0.72703	94.91727	17.76736	68.77335
KW7	1.27190	1.20047	4.51945	0.80127	112.48418	4.30337	57.58783
**Max.**	2.91563	5.12761	5.41431	1.83362	179.19951	22.75932	77.76660
**Min.**	0.00000	0.00000	2.06470	0.00000	55.11192	0.00000	53.08914
**Mean**	1.29583	1.96422	3.64861	1.05082	103.99982	4.03793	69.13433
