# Supplementary material for: Association Mapping for Evaluation of Population Structure, Genetic Diversity, and Physiochemical Traits in Drought-Stressed Maize Germplasm Using SSR Markers
Source: Plants (Basel). 2023 Dec 7;12(24):4092. doi: 10.3390/plants12244092 (PMC10747078; doi:10.3390/plants12244092)
Supplement: Supplementary file 1 [file plants-12-04092-s001.zip › Table S3.pdf]

Table S3. Maize-inbred lines and F_1 hybrids developed at the Maize Experimental Station.

Entry No.	Inbred line	Parent for F1 varieties
1	11BS8016-7	Gangwonchal 43 ♀
2	12BS5076-8	Gangwonchal 43 ♂
3	12S8052	Gangwonchal 34 ♂
4	14S8025	Gangwonchal 58 ♂
5	15RS8039	Gangwonchal 51 ♀
6	15RS8056	Gangwonchal 51 ♂
7	15RS8002	Gangwonchal 57 ♀
8	15S8021-3	Gangwonchal 48 ♀
9	16CLP23	Saekchalgyo 109 ♀
10	16CLP40	Saekchalgyo 105 ♀
11	17CS5047	Saekchalgyo 105 ♂
12	16S8068-9	Gangwonchal 48 ♂
13	17CS8006	Saekchalgyo 109 ♂
14	17CS8067	Saekchalgyo 56 ♀
15	17YS6032	Gangwonchal 60 ♀
16	17YS8003	Gangwonchal 60 ♂
17	GP3	Oryun2ho ♂
18	GP5	Oryun2ho ♀
19	HCW1	Cheongchunchal ♀
20	HCW2	Cheongchunchal ♂
21	HCW3	Hongmichal ♀
22	HCW4	Hongmichal ♂
23	HCW5	Mihongchal ♂
24	HF12	Dreamok ♂
25	HF22	Dreamok ♀
26	HW1	Dumechal ♀
27	HW10	Heugjeom2ho ♀
28	HW11	Arichal ♂
29	HW12	Gangwonchal 34 ♀, Arichal ♀, Jangsuchal ♀
30	HW15	Jangsuchal ♂
31	HW16	Goldchal ♀
32	HW17	Goldchal ♂
33	HW18	Gangwonchal 57 ♂, Mihyeonchal ♀
34	HW19	Mihyeonchal ♂, Bunongchal ♂
35	HW3	Mibaek2ho ♂, Mibaekchal ♀, Saekchalgyo 56 ♂
36	HW4	Mibaekchal ♂
37	HW7	Miheugchal ♀, Heugjeom2ho ♂
38	HW8	Miheugchal ♂
39	HW9	Gangwonchal 46 ♂, Mibaek2ho ♀, Mihongchal ♀, Bunongchal ♀
40	KL103	Heugjeomchal ♀
41	KW7	Dumechal ♂, Heugjeomchal ♂
